# Efficacy of Biofilm Removal on the Dental Implant Surface by Sodium Bicarbonate and Erythritol Powder Airflow System

**DOI:** 10.1055/s-0044-1779424

**Published:** 2024-03-31

**Authors:** Patr Pujarern, Arthit Klaophimai, Parinya Amornsettachai, Woraphong Panyayong, Boontharika Chuenjitkuntaworn, Dinesh Rokaya, Suphachai Suphangul

**Affiliations:** 1 Department of Advanced General Dentistry, Faculty of Dentistry, Mahidol University, Bangkok, Thailand; 2 Department of Oral Microbiology, Faculty of Dentistry, Mahidol University, Bangkok, Thailand; 3 Department of Prosthodontics, Faculty of Dentistry, Zarqa University, Zarqa, Jordan

**Keywords:** dental implants, peri-implantitis, mechanical debridement, air abrasion therapy, erythritol, sodium bicarbonate, biofilm removal

## Abstract

**Objective** Peri-implantitis is a common complication in implant therapy and it is one of the main contributing factors to implant failure. This can be prevented by regular maintenance with mechanical debridement. One of the recent mechanical debridement methods is air abrasion therapy using different abrasive powders. This study aimed to evaluate the two common abrasive powders of different sizes (sodium bicarbonate and erythritol) for their biofilm cleaning efficacy on dental implant surfaces.

**Materials and Methods** In an *in vitro* setting, a total of 33 implants were divided into three groups: Group 1 ( *n* =11) = no treatment; group 2 ( *n* = 11) = air abrasion therapy treated group using a sodium bicarbonate powder (AIRFLOW Powder Classic Comfort, EMS Electro Medical Systems, Nyon, Switzerland); and group 3 ( *n* = 11) = air abrasion therapy treated group using an erythritol powder (AIRFLOW Powder Plus, EMS Electro Medical Systems, Nyon, Switzerland). The implants in each group were subjected to biofilm formation, and group 2 and group 3 were treated with air abrasion therapy of two different powders having different sizes with the same settings. The particle sizes were sodium bicarbonate (40 µm) and erythritol (14µm). The surface characteristics of the dental implants in three groups were studied from a digital camera and under the scanning electron microscope at different magnifications. The comparison of biofilm-removal efficacy between the three groups was performed by using a one-way analysis of variance with post-hoc Dunnett's T3 test. A *p* -value less than 0.05 was chosen to indicate statistical significance.

**Results** There were no statistical differences ( *p* > 0.05) between the two powder-treated groups for the biofilm cleaning efficacy. However, both groups showed significantly better biofilm-cleaning efficacy than the control group ( *p* < 0.05).

**Conclusion** This suggests that both powders are effective in removing biofilm from the implant surface under ideal conditions. However, there was no clear distinction between the cleaning potential of the two powders, as both performed in a similar manner.

## Introduction

Dental implants are widely used in dentistry for prosthetic rehabilitation. 1 2 3 4 Peri-implantitis, a common complication in implant dentistry, immensely affects osseointegration and is one of the major causes of implant failure. 5 6 7 Peri-implantitis is defined as an irreversible pathological condition of the peri-implant tissues exhibiting inflammation of the mucosa and progressive loss of bone support. 5 6 8 There is a wide prevalence of peri-implantitis ranging from 10 to 31%. 9

For peri-implantitis, mechanical debridement is commonly used to prevent and remove biofilm and plaque while minimizing damage to the implant. 7 10 11 Air abrasion or polishing therapy is commonly used in mechanical debridement, and provides great accessibility and cleaning efficacy, with only a modest change to the implant surface. 6 12 13 Air polishing devices are as effective as conventional treatments for clinical and microbiologic outcomes. 14 The main advantage of the use of air polishing devices in supportive periodontal therapy seems to be their ability to efficiently remove biofilm, without causing damage to the periodontal soft tissues or tooth and root structure. In addition, air polishing devices, however, present improved antimicrobial results and are safer, faster, and more comfortable options for patients undergoing supportive periodontal therapy. 15

For air abrasion, different powders are used and with the recent introduction of erythritol (ERY) powder, air abrasion therapy is becoming increasingly popular. ERY possesses great cleaning attributes, minimal surface alteration, and biofilm-inhibiting properties. 16 17 In addition, ERY may be less abrasive than previously used ubiquitous powders, like sodium bicarbonate (SB), having a particle size that is four times smaller. 12

The difference in particle size of the powders used for the air abrasion raises another question regarding biofilm removal efficacy between the two powders, as the bigger powder can remove biofilm more efficiently. 12 18 19 Therefore, an investigation was done to evaluate whether SB and ERY show any significant differences in biofilm removal, to determine which powder should be regularly used, with the presumption that SB with its larger particle size, should have better biofilm removal capability than ERY.

## Materials and Methods

### Saliva Collection and Processing

The study was conducted in accordance with the Declaration of Helsinki. This study protocol was granted exemption from the Ethical Committee of the Faculty of Dentistry */* Faculty of Pharmacy, Mahidol University as this study does not involve humans or animals. Saliva samples were taken from a single investigator. The investigator was given paraffin wax and a test tube and instructed to chew the paraffin wax to stimulate saliva production and collect it. The saliva sample was then centrifuged by using a centrifuge (Centrifuge 5804 R, Eppendorf AG, Hamburg, Germany) at 5,000 rpm for 15 minutes. The supernatant was then transferred to another container and diluted with distilled water to obtain a 50% concentration. A pipette was used to extract 1 mL of the supernatant and placed it into a test tube containing the implant sample. The implants were left in the saliva solution for 24 hours at 37 degrees Celsius.

### Subgingival Bacterial Collection and Processing

A subgingival plaque sample was collected by the same investigator using a periodontal curette at the distal site of lower molars. The sample was then inoculated in an anaerobe basal broth and placed in an anaerobic jar (AnaeroPack, Mitsubishi Gas Chemical Co., Tokyo, Japan) to incubate for 24 hours in an anaerobic chamber. After 24 hours, 10 mL of the anaerobe basal broth was adjusted to 0.5 McFarland concentration by using a densitometer device (DEN-1 McFarland Densitometer, Biosan, BS-050102-AAF, Latvia).

### Biofilm Formation on the Dental Implants

A total of 33 implants were selected and studied in this study taking the reference of sample size from some previous studies in dental implants. 20 21 22 The dental implants used in this study were bone-level implants of size 4.0 × 8.0 mm (Dentium SuperLine II, Dentium Co., Suwon, South Korea). The implants were divided into 3 groups: Group 1 ( *n* =11) = no treatment, group 2 ( *n* =11) = SB, and group 3 ( *n* =11) = ERY. The particle sizes were SB (40 µm) and ERY (14 µm).

The coated implants were placed in the wells of a 48-well plate. One-thousand microliter of the 0.5 McFarland anaerobe basal broth was then added to each well containing an implant. The 48 well-plate was placed in an anaerobic jar (AnaeroPack, Mitsubishi Gas Chemical Co., Tokyo, Japan) and left to incubate in an anaerobic chamber for 48 hours. After the incubation period, the implants were moved into another 48-well plate and washed three times by using a 600 µL solution of phosphate buffered saline (PBS). The implants were left to dry inside a sterile cabinet, with the blower turned on.

### Airflow and Optical Density Analysis

Once dried, the implants were randomly treated as follows:

Group 1: No treatment.
Group 2: Air abrasion therapy treated group using a SB powder (AIRFLOW Powder Classic Comfort, EMS Electro Medical Systems, Nyon, Switzerland).
Group 3: Air abrasion therapy treated group using an ERY powder (AIRFLOW Powder Plus, EMS Electro Medical Systems, Nyon, Switzerland).

The treated implants were gently cleaned using the AIRFLOW Handpiece of the AIRFLOW Prophylaxis Master for 15 seconds using the same setting of power level 5, with the distance between the handpiece and implant surface of approximately 2 mm. While performing the treatment, each sample was manually rotated by hand. The implants were then left to dry in a sterile cabinet with the blower turned on.

After being dried, all the implants were placed into the wells of a 48 well-plate. A 170 µL of crystal violet 0.5% concentration was added to each well for 15 minutes, then all implants were moved to a 48 well-plate, rinsed 3 times by using a 600 µL solution of PBS, and left to dry in the same cabinet. Once dry, some random samples from each group were investigated using a stereomicroscope. The implants were then placed into a 48 well-plate along with a 600 µL of 20% acetone in an ethanol solution. The 48 well-plate was then put on a shaker (Biosan, TS-100, Bunkyo, Japan) at a medium setting for 15 minutes. Finally, two 100 µL of each sample were pipetted into a 96 well-plate to be analyzed by the microplate reader (Synergy H1, BioTek, Vermont, United States). The groups were compared for their biofilm-cleaning efficacy.

### Surface Characterization

The surface characteristics of the dental implants in three groups were studied from a stereomicroscope. In addition, the implant surfaces were studied under the scanning electron microscope (JSM-6610LV, Jeol USA Inc., Massachusetts, United States) at different magnifications, and images were taken.

### Statistical Analysis

Data analysis was performed with a statistical software program (SPSS Statistics version 28.0.1.1, IBM Corporation, Chicago, Illinois, USA). The descriptive statistics of the data were analyzed, with the means and standard deviation being used. The data was tested for normality by means of the Shapiro–Wilk test. The comparison of biofilm-removal efficacy between the three groups was performed by using a one-way analysis of variance with post-hoc Dunnett's T3 test. A *p* -value less than 0.05 was chosen to indicate statistical significance.

## Results

### Surface Characteristics

**Fig. 1:**
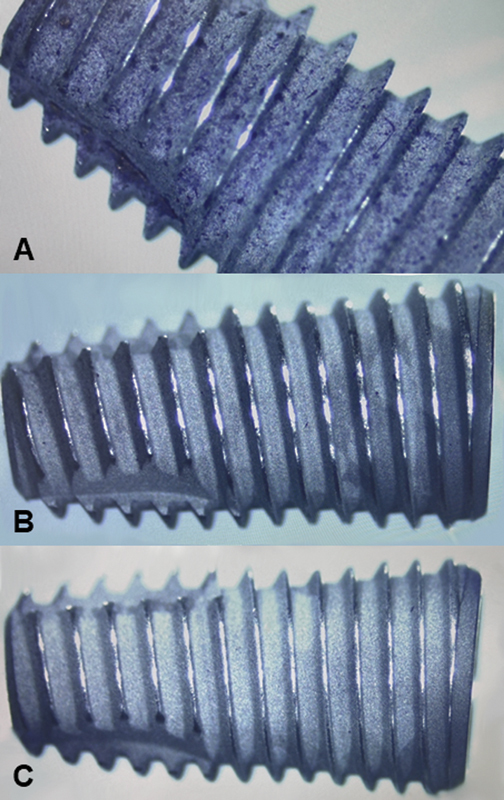
Microscopic images of the three study groups. Implant with no treatment (control) ( **A** ). Implant treated by sodium bicarbonate powder (AIRFLOW Power Classic Comfort) ( **B** ). Implant treated by erythritol powder (AIRFLOW Power Plus) ( **C** ).

After each specimen was treated and dyed with crystal violet, the remaining biofilm on the implant could then be seen as small purple spots. Fig. 1 shows the surface characteristics of the dental implants of three groups studied from a stereomicroscope. The microscopy images of the control group surface revealed the textured microstructure of the samples and biofilm can be seen covering the entirety of the implant ( Fig. 1A ). In both the SB-treated group ( Fig. 1B ) and the ERY-treated group ( Fig. 1C ), only a small number of purple spots can be seen on the implant surface. The surface structures in groups 2 and 3 show similar topography under the microscope.

**Fig. 2:**
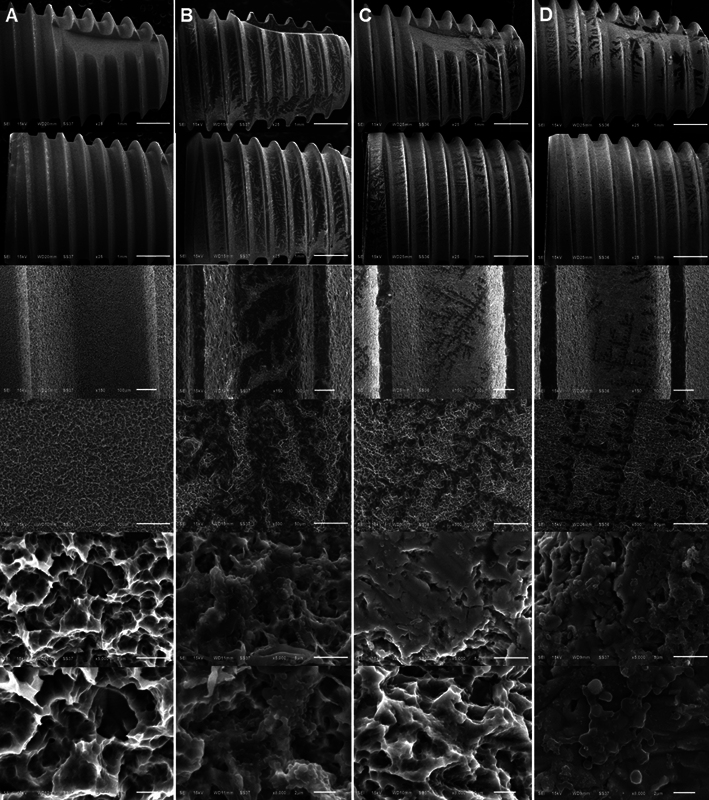
Scanning electron microscope images of the three study groups. Untreated implant ( **A** ). Implant with no treatment (control) ( **B** ). Implant treated by sodium bicarbonate powder (AIRFLOW Powder Classic Comfort) ( **C** ). Implant treated by erythritol powder (AIRFLOW Powder Plus) ( **D** ).

Fig. 2 shows the results of surface characteristics of the untreated dental implants and various groups studied under the scanning electron microscope in different magnifications. The untreated implant surface has smoother topography compared to the other groups. The micrographs of the implant surfaces of the three groups showed the textured microstructure of the samples. No marked differences between the groups were identified between groups 2 and 3 in various magnifications ( Fig. 2C and 2D ).

### Biofilm on Implant Surfaces

The opacity density (OD) of each sample represents the remaining biofilm on the untreated implants (control), implants treated using SB powder air abrasion system (group 2), and finally those implants treated using ERY powder air abrasion system (group 3). The OD of each sample was calculated by averaging two readings of the same specimen and subtracted by the OD of the negative control (0.0635).

**Fig. 3:**
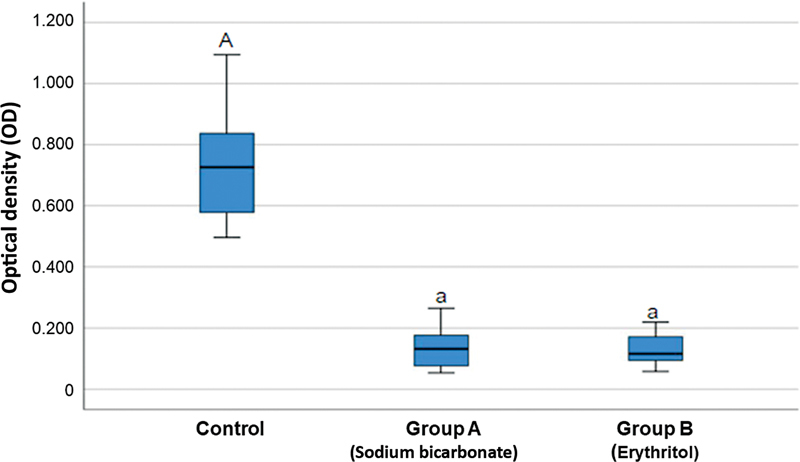
Box plot representing the opacity density (OD) of the three study groups. Implant untreated (control) ( **A** ). Implant treated by sodium bicarbonate powder (AIRFLOW Powder Classic Comfort) (B). Implant treated by erythritol powder (AIRFLOW powder Plus) ( **C** ). Letters were used to indicate any statistical differences; A and a show statistically different, while a and a show no statistical difference.

The mean OD of the control ( Table 1 ) was the highest at 0.728 ± .188, while the mean of group 2 and group 3 was at 0.130 ± 0.068 and 0.129 ± 0.049, respectively ( Fig. 3 ). Post-hoc analysis was performed using Dunnett's T3 and revealed that there was no statistical difference ( *p* > 0.05) between the groups. However, both groups showed significantly better biofilm-cleaning efficacy than the control group ( *p* < 0.05).

**Table 1:** Descriptive statistics of the study groups

Group	Mean	Standard deviation	Minimum	Maximum
Group 1 (control)	0.72	0.188	0.497	1.0955
Group 1 (sodium bicarbonate)	0.13	0.068	0.053	0.265
Group 2 (erythritol)	0.129	0.049	0.0575	0.2195

Group 1: No treatment. Group 2: Air abrasion therapy treated group using a sodium bicarbonate powder (AIRFLOW Powder Classic Comfort, EMS Electro Medical Systems, Nyon, Switzerland). Group 3: Air abrasion therapy treated group using an erythritol powder (AIRFLOW Powder Plus, EMS Electro Medical Systems, Nyon, Switzerland).

## Discussions

The mouth is a congenial environment for the growth of microorganisms by exhibiting an ideal nonshedding surface. Dental plaque happens to be a diverse community of microorganisms found on the tooth surface. 23 Implant biofilm can lead to infection at two levels: mucosal level (peri-implant mucositis and peri-implantitis) that is explained as an inflammatory lesion affecting the supporting tissues. 24 The peri-implant diseases immensely affect the osseointegration and success rate of a dental implant, eventually leading to implant failure that is peri-implantitis. 5 6 8 Hence, the management of peri-implant infections aims at the reduction in inflammation, pathogenic bacteria, and probing depths. An important method to prevent and treat peri-implantitis is regular maintenance using air abrasion therapy. Dental implants have rough surfaces and implants with even moderate-roughness surfaces accumulated more bacterial biomass and a significantly higher number of pathogenic bacteria *(Fusobacterium nucleatum* and *Aggregatibacter actinomycetemcomitans)* , when compared to implants with minimal-roughness surfaces, within a similar biofilm structure. 25

There are various cleaning methods mentioned in the literature for peri-implantitis such as airflow, small brush, and electrolytic. 7 26 27 There are chemical, pharmacological products, and herbal products that can be used in the treatment of peri-implantitis. 27 28 Mechanical debridement using air abrasion or polishing therapy provides great efficacy in cleaning the implant surface. 6 12 13

The powders we tested in this study (SB and glycine [GLY]) are the two most used in air polishing, where GLY was shown to be the superior choice. 29 This was mainly due to GLY having greater efficiency in biofilm removal, its antibacterial properties, and minimal damage to the implant surface. 29 30 31 However, some studies showed a direct correlation between using a larger particle size with higher biofilm removal. 19 31 This would lead to the notion that a sodium bicarbonate powder air abrasion system (SB), in an ideal condition, should remove biofilm better than a GLY powder air abrasion system (GLY). Recently, the erythritol powder air abrasion system (ERY) was introduced for use in air abrasion therapy with substantially better results in terms of minimal surface changes and efficiency, as well as its ability to inhibit biofilm and plaque formation similar to that of GLY. 17 18 32 As a result, speculation was made as to which powder should become the gold standard in air abrasion therapy, as both GLY and ERY have smaller particle sizes (with ERY being the smallest), but both have similar antimicrobial properties. 17 19 Therefore, if ERY can be shown to remove biofilm with the same capacity as SB, then it would be rational to make ERY the clinically best overall choice for use in air abrasion therapy. This led to the present study, where we compared the efficacy of biofilm removal on the implant surface by using both SB and ERY powder airflow systems with the expectation that SB with a bigger particle size would be more effective in biofilm removal.

In this study, both SB and ERY proved to be effective in biofilm removal on implant surfaces, as both groups when compared to the control group were statistically different. However, when comparing SB and ERY, no statistical differences were seen. This suggested that both SB and ERY were effective in removing biofilm from the implant surface, which is in line with previous studies; however, the difference between the two is too less. 19 33 Our study results indicated that particle size might not make a difference, even though SB particles are almost four times larger than ERY. 19 31 Another reason might be that crystal violet staining may not be sensitive enough to differentiate minute remaining biofilm after each treatment, thus a similar remaining biofilm density was shown. Research done by Matsubara et al 19 suggested that larger particle size is directly correlated to an increase in biofilm removal and the smaller particles resulted in less effective cleaning capacity than larger particles; however, the results showed less topographical change of the surface when using smaller particles when compared to the larger particles. Given the fact that their study model used implants covered with ink instead of biofilm-like in the present study, with ink having different means of attaching to the implant, it might be the reason behind the difference in cleaning capacity. 19 Other studies have shown that larger particle size can remove biofilm more effectively than smaller particles, while other study findings contradict the idea suggesting that the cleaning performance was the same. 18 Another study suggested that smaller particles are gentler on the soft tissue and cause less damage to the gingiva compared to larger ones. 31 Therefore, with SB and ERY having nearly identical cleaning abilities, evidence suggests the use of a smaller particle powder might be better, as smaller particles have been shown to cause less damage to the peri-implant tissue.

Nevertheless, it has been established that air abrasion therapy performs better than other instruments used in mechanical debridement, as it can access areas of the implant that are limited to traditional mechanical instruments. 18 Additionally, as shown in Fig. 1B and C , nearly all the initial biofilm was removed even on the hard-to-reach areas of the thread and valley surface. This is similar to the study by Cha et al 12 and the air abrasion system can reach hard to accessed areas of the implant better than other methods. Moreover, research suggests that when comparing air abrasion therapy to other mechanical debridement methods, air abrasion therapy is proven to be less damaging to the implant surface. 12 34 35

It is found that various cleaning instruments on dental implants may cause undesired surface alterations. Meschenmoser et al 36 recommended that the titanium curet and the air abrasive system can only be recommended with restrictions. The steel curet and the ultrasonic system proved to be unsuitable for cleaning titanium implants. Some studies have been done to study the surface alterations following the implant surface cleaning. 37 38 Schmage et al 37 studied the effects of various implant cleaning instruments on four implant surfaces (polished, grit-blasted, acid-etched, and acid-etched/grit-blasted). They found Ra and Rz values were significantly lower on grit-blasted/acid-etched implant surfaces following the use of the Sonic-Flex clean with prophylaxis brush and the plastic curette compared to Satelec ProphyMax with Periosoft curette. The Ra and Rz values for the acid-etched surfaces and Ra, Rz, and Lr values for the polished and the grit-blasted surfaces showed no significant differences between the different cleaning methods or cleaning instruments compared to the control. They concluded that the cleaning effect and the implant surface alterations were strongly dependent on the implant cleaning method used. Similarly, Augthun et al 38 studied the effect of cleaning procedures on the surfaces of three implant types (plasma sprayed, hydroxyapatite-coated implants, and smooth titanium surface) using a scanning electron microscope where they studied six different hygiene measures: plastic curet, metal curet, diamond polishing device, ultrasonic scaler, air-powder-water spray with sodium hydrocarbonate solution, and chlorhexidine 0.1% solution rinse. They found that the air-powder-abrasive system, chlorhexidine rinse, and curettage with a plastic instrument caused little or no surface damage in all but the hydroxyapatite-coated fixtures. Only the sodium hydrocarbonate spray resulted in a clean fixture without damage to the implant surface.

In dental implants, special treatment was applied on the surface to create favorable surface roughness that would not only increase osseointegration but also reduce biofilm formation. 35 Thus, through regular maintenance of the dental implants, it is ideal to utilize a mechanical debridement method least damaging to the implant surface. 12 33 In a previous study by Matsubara et al 19 and Cha et al, 12 they compared the different air abrasion powders using a scanning electron microscope and they found that SB was seen to be the most damaging, while both GLY and ERY barely altered the implant surface. 12 19 39 Other studies also indicate that SB creates a crater-like defect on the implant surface that could potentially facilitate plaque accumulation and affect osseointegration. 39 However, surface changes of the titanium implant after air abrasion therapy were not documented in this experiment, as surface topographical changes cannot be seen by the stereomicroscope. This means there might be microscopic changes to the surface of the implant that differ between the two powder-treated groups; however, evidence suggests the use of smaller particle sizes, as they are shown to cause minimal damage to the implant surface. 12 19 39 40

This study aimed to differentiate the cleaning efficacy between large and small particle airflow systems. The visualization of the implant surface is limited in this study, as only the stereomicroscope was used. The use of a scanning electron microscope would be better to visualize any protocol effect on the surface of the implant. Without any statistical difference seen between the two treated groups and various evidence from previous studies led to the consensus that favors the use of smaller particle size, in this case, the ERY airflow system. However, mechanical debridement with air abrasion therapy was conducted in an ideal condition within an *in vitro* setting. This limits the extent of clinically concluding which powder is superior, due to the fact in an actual clinical setting, ideal conditions and accessibility generally cannot be achieved. Therefore, in clinical settings different particle sizes might make a difference in cleaning, as the gingival crevices depth and width, implant location, and microflora all vary among different individuals. Also, the plaque colony is taken from a single individual, thus the diversity in the community could not reflect that of an entire population, meaning the thickness and stickiness of the colony could differ from that of actual clinical settings. In addition, the method of using crystal violet staining and measuring the OD did not differentiate between dead and live colonies; thus it is not easy to determine the antimicrobial property of the method used.

For further future studies, better replication of the clinical setting should be considered to replicate real-life situations more accurately, and ideally, the experiment should be conducted in an *in vivo* setting. Furthermore, more surface characterization can be done to study the surface charge of each implant, as well as apply a better method to quantify the efficacy and efficiency of the two powders to fully visualize the differences. However, even with limitations due to the *in vitro* setting, further studies should be conducted with a setting that fully replicates the clinical use of the airflow system, to determine both the risks and benefits of this novel mechanical debridement procedure more accurately.

## Conclusion

Mechanical debridement using air abrasion or polishing therapy provides great efficacy in cleaning the dental implant surface. Both SB and ERY powder proved to be effective in removing biofilm from implant surfaces when performed under ideal conditions. However, no clear difference between the cleaning efficacy of the two powders was found, as both seem to perform at a similar level.
